# Towards Physiologically and Tightly Regulated Vectored Antibody Therapies

**DOI:** 10.3390/cancers12040962

**Published:** 2020-04-13

**Authors:** Audrey Page, Floriane Fusil, François-Loïc Cosset

**Affiliations:** CIRICentre International de Recherche en Infectiologie, Univ Lyon, Université Claude Bernard Lyon 1, Inserm, U1111, CNRS, UMR5308, ENS Lyon, 46 allée d’Italie, F-69007 Lyon, France; audrey.page1@ens-lyon.fr (A.P.); floriane.fusil@ens-lyon.fr (F.F.)

**Keywords:** adoptive transfer, antibody, cell engineering, checkpoint inhibitors, gene editing, neutralization, reprogramming, viral vectors

## Abstract

Cancers represent highly significant health issues and the options for their treatment are often not efficient to cure the disease. Immunotherapy strategies have been developed to modulate the patient’s immune system in order to eradicate cancerous cells. For instance, passive immunization consists in the administration at high doses of exogenously produced monoclonal antibodies directed either against tumor antigen or against immune checkpoint inhibitors. Its main advantage is that it provides immediate immunity, though during a relatively short period, which consequently requires frequent injections. To circumvent this limitation, several approaches, reviewed here, have emerged to induce in vivo antibody secretion at physiological doses. Gene delivery vectors, such as adenoviral vectors or adeno-associated vectors, have been designed to induce antibody secretion in vivo after in situ cell modification, and have driven significant improvements in several cancer models. However, anti-idiotypic antibodies and escape mutants have been detected, probably because of both the continuous expression of antibodies and their expression by unspecialized cell types. To overcome these hurdles, adoptive transfer of genetically modified B cells that secrete antibodies either constitutively or in a regulated manner have been developed by ex vivo transgene insertion with viral vectors. Recently, with the emergence of gene editing technologies, the endogenous B cell receptor loci of B cells have been modified with the clustered regularly interspaced short palindromic repeats (CRISPR)/CRISPR-associated endonuclease (Cas-9) system to change their specificity in order to target a given antigen. The expression of the modified BCR gene hence follows the endogenous regulation mechanisms, which may prevent or at least reduce side effects. Although these approaches seem promising for cancer treatments, major questions, such as the persistence and the re-activation potential of these engineered cells, remain to be addressed in clinically relevant animal models before translation to humans.

## 1. Introduction

Currently, cancers remain a highly significant health burden, causing around 10 million deaths per year, which represent the second leading cause of death worldwide according to the World Health Organization, after cardiovascular diseases. Therapeutic strategies that are routinely used in the clinic mainly rely on chemotherapy, radiotherapy and surgery. However, these treatments are not efficient enough for some cancers, either to cure the disease or to prevent recurrences, highlighting the urgent need for novel, efficient, safe, cost-effective and less-invasive approaches. In this context, immunotherapy represents a promising alternative for cancer clearance, through the direct modulation and education of the patient’s immune system to eradicate cancerous cells. Although the concept of immunotherapy is not new—since the end of the 19th century, the inoculation of bacteria or live cells into the tumors was already considered to treat malignancies—the number of immunotherapy trials to fight cancer have exploded over the past decades [1,2,3].

Two main therapeutic strategies have been developed to confer protective immunity against cancers. The first one, vaccination or active immunization, relies on exposing patients to tumor’ components in order to build up an immune memory, for example, through the infusion of tumor lysates or of dendritic cells pulsed with tumor antigens. Although most approaches were specifically designed to enhance CD8+ T cell response, the protective efficacy of currently used vaccines is also mediated by the induction of antibodies (Ab) through B cell mobilization, both cellular and humoral responses conferring long-lasting immunity [4,5]. However, it takes several weeks or months and several injections to create a vaccine-induced immunity. In addition, optimal protection is rarely achieved in the case of cancers and immune defenses in elderly people, a population highly susceptible to cancers, are weaker, making active immunization even more challenging.

An alternative approach, called passive immunization, consists in the administration of exogenously produced protective monoclonal Abs (mAbs). Because it does not require previous immunization and generation of immune memory, passive immunization constitutes a therapeutic approach that can hopefully control a disease when it has already occurred by providing immediate immunity. Several types of host molecules can be targeted by the injected protective mAbs.

First, these antibodies may target specific surface molecules that are expressed primarily and, ideally, only on tumor cells. However, such tumor-specific antigens are rarely known or vary among patients. Consequently, antigens that are present on tumor cells but also on certain normal tissues, called tumor-associated antigens (TAAs) are often used as disease biomarkers. TAAs can be divided into different classes, depending on their origin and their molecular structure. Among them are 1) some “cluster of differentiation” antigens, such as CD20 for non-Hodgkin lymphoma, CD30 for Hodgkin lymphoma, CD33 for acute myelogenous leukemia, and CD52 for chronic lymphocytic leukemia), 2) vascular targets, such as vascular endothelial growth factor (VEGF), and 3) several growth factor receptors, such as human epidermal growth factor receptor 2 (HER 2). TAA-targeted Abs can operate through direct or immune-related killing of tumor cells. Indeed, the Ab constant region mediates interactions with complement proteins as well as with Fc receptors expressed on many immune cells, including neutrophils, macrophages, natural killer cells and B cells, which triggers effector functions such as complement-dependent cytotoxicity (CDC), antibody-dependent cell cytotoxicity (ADCC) or antibody-dependent cellular phagocytosis (ADCP). In addition, antibodies may act directly, notably by blocking pathologic signaling cascades or soluble factors, as well as by inducing apoptosis.

Second, antibodies that are specific to some surface immune regulatory molecules called immune checkpoint have been developed for cancer treatment. Indeed, since the tumor microenvironment is globally immunosuppressive, the response to treatment is reduced, which facilitates tumor growth. For example, PD-L1 and CTLA-4 are well-characterized immune checkpoint inhibitors that are overexpressed in many cancer cells and promote immune escape. Antibodies against PD-L1 and CTLA-4 have proven effective to treat some cancers in clinical trials by blocking the immunosuppressive interaction with their receptors expressed on immune cells. Alternatively immune checkpoint activators, such as immunostimulatory anti-OX40 antibodies, have been developed to increase the activation and effector functions of the immune system against cancer cells [6,7]. Yet, these strategies require some improvements especially to predict patients who can respond [8].

A major advantage of passive antibody infusion is that it is possible to engineer the injected Abs to potentiate their therapeutic properties. One option consists in engineering the variable regions to target several antigens. Accordingly, bispecific Abs recognizing two different TAAs or, alternatively, a TAA and a checkpoint inhibitor were developed, in order to enhance the precision of targeting and the effect of Abs, respectively [9,10]. Recently, a tri-specific antibody targeting CD38, CD3 and CD28 was designed to activate T cells against B cell malignancies [11]. Depending on the antibody isotype or even on the immunoglobulin subtypes chosen, effectors functions will vary. For instance, IgG (1–4) subtypes exhibit different capacities to activate the complement pathway and to bind immune cell receptors. As an alternative option, the constant region of antibodies can also be modified to modulate their ability to induce ADCC and CDC. This can be achieved by mutating the amino acids allowing the interaction with Fc receptors or with complement molecules [12,13,14]. For instance, Fc-silent immunoglobulin variants have been constructed to extend antibody half-life or to suppress Fc-mediated effector functions, when not mandatory for therapeutic efficacy [14,15]. Alternatively, antibodies can also be engineered to carry drugs, which will be delivered to the right location.

However, passive infusion of monoclonal antibodies has still several limitations. The main drawback often associated to such therapies is that it only provides short-term protection, owing to the relatively short half-life of Abs. Furthermore, except when using immune checkpoint blockers that can enhance pre-existing anti-tumor immunity, no memory responses are usually induced following monoclonal antibody infusion. Consequently, frequent injections of therapeutic Abs for several months or even years are required, generating elevated costs and inconvenience to patients due to frequent ambulatory care. Furthermore, such antibodies are often intravenously injected at doses well-above the physiological concentrations to reach clinical efficacy, generating systemic side effects, such as cardiac and renal failure or cytokine storms [16,17]. Hence, new active immunization strategies have been developed to circumvent these limitations and to induce antibody secretion at physiological doses in vivo, either through the infusion of engineered antibody-producing cells or through in situ gene modification of endogenous cells.

Here, we review the main approaches that have been undertaken over the past years to directly produce ectopic antibodies in vivo.

## 2. Active Immunotherapy Approaches for Antibody Secretion (Vectored Immunoprophylaxis)

Vectored immunoprophylaxis allows the secretion of transgenic antibodies in vivo after in situ gene transfer upon vector infusion [18]. Although this strategy was initially investigated in preclinical studies for chronic diseases induced by infectious pathogens (e.g., human immunodeficiency virus, HIV [19] and simian immunodeficiency virus, SIV [20], hepatitis C virus, HCV [21]), it was more recently translated to other pathologies, such as cancers.

### 2.1. General Notions of Vector Design

Antibodies are formed by two chains: a heavy chain, encoded by one locus, and a light chain, which can be either of κ or λ type, encoded by two different loci. Antibodies harbor two light chains and two constant chains. The association of the variable regions of the light and heavy chains forms the paratope, which interacts with the target antigen, while the constant regions of the heavy chain harbor effector functions.

Consequently, the minimal sequences required to encode immunoglobulins are complex and already quite long (around 2.5 kb). In addition, promoters driving the expression of the light and the heavy chains were initially co-inserted in constructs allowing Ab gene transfer, which made this approach even more challenging because of the limited packaging capacity of the vectors. For example, adeno-associated virus (AAV)- and lentivirus-derived vectors, which are commonly used gene delivery vectors, have limited packaging capacities, of around 5 kb for AAV and 10 kb for lentiviral vectors (LV). Yet, the introduction between the two Ab chains of an IRES sequence or of a 2A peptide, which induces ribosomal skipping, allows that only one promoter is required [22,23,24,25,26,27].

Globally, several modes of infusion have been tested depending on both the vector type and the targeted tissue in preclinical mouse models. Frequently used infusion routes include intraperitoneal and intravenous delivery, which can promote systemic response. Yet, for solid cancers, the vectors can be directly injected into tumors to enhance their efficacy locally [28]. For instance, intracranial delivery was efficient to treat breast cancer brain metastases [29,30]. Furthermore, oncolytic viruses that have tropism toward tumors and that exhibit a lytic effect against tumor cells can also be suitably engineered to both deliver transgenes in restricted locations and concomitantly exploit their antitumoral properties. For instance, recombinant Semliki Forest virus and influenza A virus, expressing respectively anti-PDL-1 antibodies and a single-chain antibody against CTLA4 have been developed [31,32]. Alternatively, vectors that do not have a natural tropism toward tumors can be engineered to target tumor cells (Appendix A) [33,34].

AAV-based vectors have a single-stranded DNA genome that can persist in an episomical manner, which leads to long-term transgene expression, particularly in non-dividing cells. On the other hand, LVs have a single-stranded RNA genome that can efficiently integrate into the host genome after reverse transcription, which can drive sustained transgene expression. Both viral vector types are highly efficient gene transfer machines and can easily be produced. Furthermore, they have been validated in numerous clinical trials, although several safety and efficacy issues still remain to be addressed. For the latter point, to circumvent Ab-mediated neutralization that could limit their efficacy, owing to the >70% AAV serotype 2 seropositivity of the population and notably T-cell mediated responses, AAV vector particles are often injected into muscles [35]. Interestingly, association of AAV vectors to extracellular vesicles or vector pegylation were shown to induce escape from neutralizing antibodies and represents an attractive gene delivery option [36,37,38].

Although, engineered viruses are nowadays a prevalent gene delivery method, plasmids encoding cDNAs can also be directly electroporated in vivo [39]. For instance, the electroporation of a plasmid encoding an antibody directed against PSMA (prostate specific membrane antigen), a TAA associated to prostate cancer, led to the production of transgenic antibodies in vivo [39].

### 2.2. Applications for Cancer Therapy

Over the past decade, vectors encoding antibodies directed against TAAs have been developed and tested in several murine cancer models, showing promising results. Most preclinical studies involving vector-mediated antibody expression have focused on colorectal cancers, which are among the deadliest cancers. Adenoviral vectors encoding antibodies directed against epidermal growth factor (EGF), p21Ras or tissue factor were shown to afford significant benefits in murine colorectal cancer models [40,41,42]. Hormone-related cancers, such as breast cancer or ovarian cancer, have also been significantly reduced in murine models [28,43], using, for example an oncolytic adenovirus vector encoding trastuzumab, a humanized specific mAb directed against HER-2 [43]. Other cancers have gained increasing attention for vectored immunoprophylaxis, notably myeloma, lung cancers and epithelial cancers, and for which vector infusion have led to delayed tumor development [44,45,46].

Alternatively, vectors have also been engineered to target the immunosuppressive microenvironment, using, for instance, anti-PD-L1 antibodies upon transduction with retrovirus- or Newcastle disease virus-based vectors, which reduced tumor growth and prolonged survival in glioma [47] and melanoma [48] murine models. Antibodies targeting other inhibitory checkpoints, such as PD-1 or CTLA4 have also driven significant improvements after vector delivery in murine cancer models [49,50,51]. Other components of the tumor microenvironment, that are not immuno-related, have been targeted by vectored immunotherapy such as VEGF, which promotes angiogenesis. The expression of a monoclonal antibody directed against VEGF induced by AAV transduction led to a reduction of tumor growth and metastasis in vivo [28,52].

Through vectored immunoprophylaxis, antibodies targeting different components, such as angiogenesis molecules, tumor antigens, and immune checkpoints, can be combined in a single infusion in order to potentiate treatment efficacy.

However, even if these strategies showed promising results on cancer regression in preclinical studies, translation to humans is still challenging, mainly for safety issues, such as pathological immune responses against the vector or the therapeutic antibody, as discussed below.

### 2.3. Immune Consequences after Vector Infusion

Preclinical studies have highlighted the numerous advantages of vectored immunoprophylaxis as compared to the passive infusion of antibodies. First, the protection is durable for several months and even years. For instance, antibodies against HIV were still detectable in the sera of monkeys 11 months after AAV inoculation [19]. Consequently, the frequency of vector administration into patients would likely be reduced, which could improve their quality of life and reduce the costs associated to monoclonal antibody therapies. Second, with passive antibody infusion, massive doses of antibodies are repeatedly injected into patients, whereas with vectored immunoprophylaxis, the antibodies are continuously delivered at low and near physiological levels. This should prevent the neutralizing anti-idiotypic immune response, which is often observed with conventional monoclonal antibody therapies. However, although their appearance was delayed, anti-idiotypic antibodies have nevertheless been detected with vectored immunoprophylaxis, which might be due to the constitutive and nonregulated expression of the therapeutic Ab [53].

On the other hand, vectored antibody immunoprophylaxis also has several drawbacks that need to be overcome before it can be fully put into clinical practice. First, immune reactions can be triggered against the vector itself, hence limiting its range of action and particularly its re-administration. Second, due to the selection pressure induced by the continuous presence of neutralizing antibodies, escape mutants of the targeted molecules, e.g., TAAs, might arise (reviewed in [54]). Thus, implementing a temporally regulated secretion of antibody might reduce this selective pressure by decreasing exposure duration, as discussed below. Third, tumor penetration by antibodies is often relatively low. The injection of the vector directly into the target tissue might increase tumor access and might also slightly reduce its systemic side effects. Finally, vector particles can be engineered to transduce specific cell types via surface engineering or to drive therapeutic Ab from specific cell types using cell-specific promoters, which can be used to restrict vector expression. This is important since some cell types might not allow the correct folding or maturation of antibodies, leading to structural abnormalities that can be recognized as foreign antigens by the immune system. The delivery route also needs to be carefully considered since intramuscular injection of Ab-expressing vector can increase the immune response against the vector and the therapeutic Ab [55].

Consequently, it is clear that the secretion of antibodies at physiological levels by specialized cells might be a more efficient approach. Toward this goal, adoptive transfer cell therapies have emerged in which cells transduced ex vivo are subsequently reinfused, likely circumventing the above-mentioned issues.

## 3. Transgenic Antibody Expression by B Cells

### 3.1. Constitutive Expression of Transgenic Antibodies by B Cells

**Figure 1:**
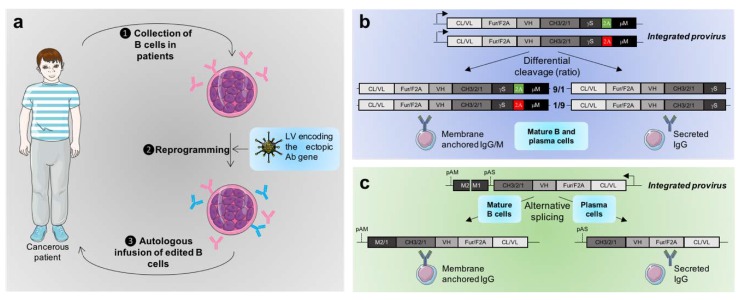
Reprogramming approach for the ectopic expression of membrane anchored and/or secreted antibodies. (**a**) Clinical approach. B cells from patients would be collected, modified, and then re-infused. Ex vivo B cell receptor insertion would be achieved by transduction with a lentiviral vector carrying the antigen-specific immunoglobulin sequence. (**b**) The molecular rheostat approach. A LV construct encoding the membrane-anchored and/or secreted form of an antigen-specific-IgG1/M driven by Igκ light chain promoter (FEEK) was generated. The constant IgG1 heavy chain (CH) and κ light chain (CL) genes were fused to the variable regions (VL and VH) of a monoclonal antibody directed against a specific antigen. Co-expression of CHs and VHs were obtained by introduction of the F2A peptide sequence. A 2A peptide was included between the secreted γ1 exon and between the μM domain. By mutating the 2A peptide, modulation of the secreted IgG and surface chimeric IgG/M BCR ratio can be achieved. (**c**) The FAM2 approach. A LV construct encoding the membrane-anchored and/or secreted form of an antigen-specific-IgG1 driven by Igκ light chain promoter (FEEK) was generated. The constant IgG1 heavy chain (CH) and κ light chain (CL) genes were fused to the variable regions (VL and VH) of a monoclonal antibody directed against a specific antigen. Co-expression of CHs and VHs were obtained by introduction of the F2A peptide sequence. Two short intronic sequences were included between the CH3 and M1 exons and between the M1 and M2 exons as well as two polyadenylation signals respectively before and after the transmembrane domains. The production of secreted or membrane-anchored immunoglobulins closely mimics the natural expression of these two distinct immunoglobulin forms, which is tightly controlled by alternative splicing and polyadenylation mechanisms during B-cell lymphopoiesis.

Although non-B cells embedded in polymer devices have been used to produce antibodies after implantation, B cells, which are natural antibody-producing cells, have focused attention in adoptive transfer approaches (Figure 1A) [56,57,58,59]. The development of GMP-closed circuits allowing both modification and expansion of immune cells ex vivo will significantly increase the availability of such treatments. In addition, vectors that can efficiently transduce B cells have recently been developed [60,61], making B cell-engineering much easier than before (Appendix B), as illustrated by a recent clinical trial involving a B cell transfer [62].

B cells derive from the differentiation of hematopoietic stem cells (HSC), which could directly be targeted to allow ectopic antibody expression by mature B cells. Demonstrating the feasibility of antibody expression after HSC transduction and differentiation into B cells, Luo et al. inserted the sequence of an anti-HIV vectored IgG with a lentiviral vector in HSCs and showed that after in vitro maturation, the descending B cell progeny could secrete the ectopic antibodies [63]. Furthermore, since HSCs give rise to all hematopoietic cells, B cell-specific promoters, such as immunoglobulin promoters and/or enhancers, can be used and/or designed in order to restrict BCR or Ab expression to this lineage [63,64,65]. However, although HSC modification can induce the expression of secreted antibodies, this approach seems to be less efficient regarding the expression of the BCR form and may not allow the expression of the membrane-anchored form of antibodies. Indeed, antibody anchorage at the membrane impairs B cell development, probably because of a disrupted tonic signal [66]. Thus, direct ex vivo B cell engineering and re-infusion may represent a more promising solution for cancer patients. For instance, B cells directed against a given TAA that were selected and expanded in vitro led to a significant decrease of tumor metastasis upon their infusion in a murine cancer model [67].

A major advantage of targeting B cells for ectopic antibody expression resides in their tropism, as these cells tend to invade inflamed areas. Paving the way, T cells, that also preferentially aggregate and proliferate at such pathological locations, have been engineered over the past decade as CAR T cells expressing chimeric antigen receptors for targeted cancer therapy with tremendous clinical results. For instance, remission for some responsive patients has been achieved with CAR T cell therapy approved for the treatment of myelomas [68]. Interestingly, CAR T cells targeting tumor antigens have been modified to also constitutively secrete inhibitory checkpoint antibodies, such as anti-PDL-1 [69] or anti-PD-1 [70] mAbs, potentiating treatment efficacy. Nevertheless, although the tropism of B cells could spatially restrict antibody secretion, their expression ultimately needs to be temporally regulated for the above-mentioned reasons, notably to reduce anti-idiotypic responses. Addressing this important issue, specific vectors have been designed to control the temporal expression pattern of transgenic antibodies.

### 3.2. Toward the Physiologically Regulated Expression of Ectopic Antibodies

The secretion of antibodies by B cells is both temporally and spatially regulated by physiological mechanisms. Indeed, during B cell maturation into plasma cells, cells switch toward an increased production of the soluble form of immunoglobulins instead of the BCR form that is predominantly expressed at earlier stages of B cell lymphopoiesis. As previously mentioned, immunoglobulins consist of two heavy chains and two light chains, which derive from three different loci, one coding the heavy chain on chromosome 14 and two encoding the light chains, λ and κ, respectively on chromosomes 22 and 2. The premessenger RNA encoding Ab heavy chains is alternatively spliced on its 3′ side by a competition process involving the splicing reaction and the polyadenylation cleavage to produce spliced mRNAs encoding the membrane anchored form of the Ab (BCR) vs. the unspliced mRNAs encoding the secreted form of the Ig protein. The ratio of spliced/unspliced mRNAs is higher in mature B cells than in plasma cells [71].

Several groups have attempted to mimic in vectored immunoprophylaxis what naturally occurs to regulate the amounts of secreted immunoglobulins. In 2012, the group of D. Baltimore developed the “molecular rheostat” system allowing the concomitant expression of both the membrane-bound and the secreted forms of a therapeutic Ab from a unique LV construct (Figure 1B) [72]. This LV construct encoded the light and heavy chain of an immunoglobulin directed against HIV with an engineered 2A peptide site inserted between the γ secreted exon from an IgG and the μM transmembrane anchor, from an IgM. Although natural 2A peptides induce 100% cleavage efficiency, this ribosome skipping efficiency can be reduced when mutations are introduced in their sequence [73]. Thus, by selecting mutations in the 2A peptide, the authors could tune the ratio of membrane-anchored vs. secreted antibodies produced by the vector. It remains to be noted that this ratio would be the same independently of the B cell maturation status. In addition, although this system allows the concomitant expression of membrane-anchored and secreted antibodies in a defined ratio, the temporal pattern of immunoglobulin secretion is not regulated. Accordingly, inducible vectors have been developed to temporally achieve controlled Ab secretion in vivo using adjustable transgene transcription via small molecules such as tetracycline, doxycycline, or rapamycin. For instance, a high level of full-length antibodies has been achieved in vivo using a rapamycin-inducible AAV vector [74]. Obviously, the level of secreted antibodies is not physiologically regulated but rather induced by the ingestion or infusion of a given molecule. Thus, the ideal system would consist in a system in which the antibodies are secreted only after the cells encounter their specific antigen in vivo, mimicking the physiological regulation.

Toward this aim, our group developed the so-called “FAM2 technology” (Figure 1C), whereby transgenic IgG1-encoding lentiviral vectors harbor both the light and heavy chain with a T2A and furin site in between [75]. The particularity of the FAM2 vector lies in the IgG intronic sequences present between the exons encoding its two transmembrane domains, M1 and M2, and between the CH3 and the M1 domains. Two polyadenylation signals, respectively positioned before and after the transmembrane domains are also present in the FAM2 vector. Thus, depending on the poly(A) signal used and on cell-specific splicing events, the transmembrane domains may or may not be retained, leading to expression of either the membranous (in B cells) or the secreted (in plasma cells) forms of the immunoglobulin, which could be monitored in vivo. Interestingly, the proportion of secreted antibodies was greater in plasma cells than in mature B cells, mimicking what naturally occurs during B cell maturation. The development of the FAM2 technology towards achieving physiological ratio will consist in optimizing the intron size between CH3 and M1 [76,77,78].

## 4. Direct Gene Editing of the Endogenous BCR

Ideally, the endogenous immunoglobulin regulation mechanisms should be used to ectopically express a therapeutic Ab. This requires new strategies to directly modify the endogenous antibody specificity by changing its variable regions without altering the constant regions.

### 4.1. General Principle

The expansion of gene-editing technologies is opening new avenues for the treatment of many diseases. Currently, there are four main classes of DNA-editing nucleases: zinc-finger nucleases (ZFNs), meganucleases, transcription activator-like effector nucleases (TALENs), and clustered regularly interspaced short palindromic repeats (CRISPR)/CRISPR-associated endonuclease (Cas-9) [79]; the CRISPR /Cas9 technology being the most widely used currently. These nucleases introduce double-strand breaks (DSBs) at their target site. DSBs are repaired either through the error-prone process of non-homologous end-joining (NHEJ) or through the error-free homology-directed repair (HDR) [80]. NHEJ repairs DSBs by adding random nucleotides to join the DNA strands, whereas HDR promotes the accurate copy of a DNA template that contains sequences homologous to the target site [80,81].

Several cell types can be targeted to change the specificity of endogenous BCR. First, HSCs and embryonic stem cells (ESCs) represent promising alternatives since as they give rise to B cells after differentiation, ensuring that all B cells descending from an edited stem cell will carry the modification. Of note, most of BCR transgenic mouse models have been created by direct modification of the heavy chain locus in ESCs, which is not feasible in a clinical setting [82,83]. Second, B cells can also be directly engineered, as the feasibility of B cells editing has already been demonstrated by targeting the CD19 locus [84].

**Figure 2:**
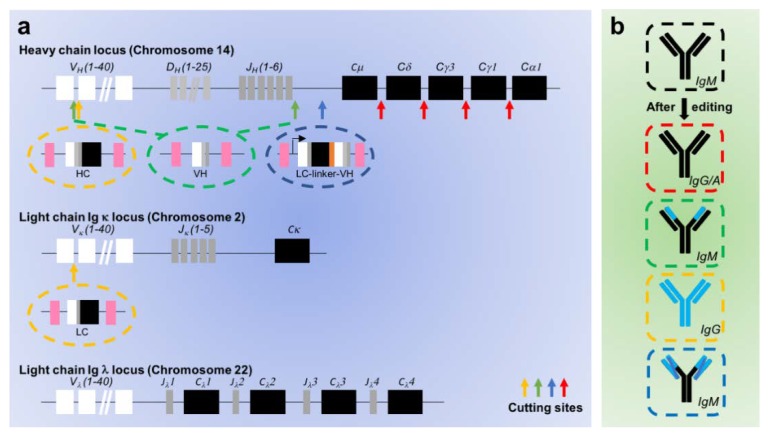
Reprogramming approach to edit of the endogenous BCR. (**a**) Cutting sites and DNA templates for BCR editing. The endogenous loci coding antibodies are displayed. The heavy chain is located on the chromosome 14 and comprises variable, joining, diversity and constant regions. The two types of light chains, κ and λ, are respectively located on chromosome 2 and 22 and comprises variable, joining and constant regions. The cutting sites targeted by different groups to edit genetically antibody loci are indicated by arrows (red [88], green [87], yellow [86], blue [85]). (**b**) Antibodies after editing. The resulting antibodies after gene editing are displayed bellow. The modified parts (different from the endogenous ones, in black) are indicated in blue.

However, editing the immunoglobulin loci is not as easy as editing the CD19 locus, since they are encoded by three different loci (Figure 2A). Several regions are present on these loci. The heavy chain locus comprises multiple variable diversity and joining regions while the light chain loci do not contain diversity regions. In addition to these regions encoding the variable part of the antibody, regions encoding the constant part are also present. During lymphopoiesis, these loci are genetically rearranged to create a Variable (Diversity) Joining (V(D)J) combination in a random manner, leading to the huge diversity of the Ab repertoire. This combination is unique for each newly generated B cell. Consequently, creating sgRNAs that can cut most B cells is a huge challenge. To circumvent this difficulty, Moffett et al. designed sgRNAs targeting the intron between the last J domain and the constant μ region (Figure 2A) [85]. Yet, directly targeting the variable or joining regions remains feasible. Indeed, two groups have shown efficient cleavage in these regions by designing sgRNAs that can bind to frequently kept V and J regions [86] or at the extremities (first V and last J domains, Figure 2) [87]. Several DNA templates have been created for homologous recombination to introduce either a new variable heavy region alone or with a new variable light region after cleavage (Figure 2) [85,87]. In addition, Greiner et al. introduced a full-length antibody chains (heavy and light) including the constant domains (Figure 2) [86]. The advantage here is that the antibody effector functions, carried on the constant domains, can be enhanced by mutating these regions before insertion. The introduced sequence(s) can be controlled by the endogenous immunoglobulin promoter or, alternatively, by an exogenous promoter, which can be added on the DNA donor template to drive the expression of the edited immunoglobulin gene.

### 4.2. Current Challenges

Although these approaches seem very promising, implementing the CRISPR/Cas9 system for BCR editing remains challenging in many regards (Table 1).

On the one hand, some limitations are due to the CRISPR/Cas9 technology itself, such as low cutting and recombination efficiencies, potent cell cycle disruption and off-target mutations to meet clinical criteria. Although off-target effects might be reduced by using a unique gRNA, a deep analysis of off-target mutations as well as several quality and safety controls should be performed before reinfusion of edited cells [90]. The delivery methods for the Cas9 protein and the gRNA are also challenging. Retroviral vectors [88], gRNA precomplexed to Cas9 protein [85,86,89], or plasmids encoding Cas9 and gRNA can be used [87]. Alternatively, combo systems, such as the “nanoblade” platform, may represent suitable alternatives to deliver the DNA donor template, the Cas9 protein and the sgRNA [91]. Finally, while the recombination efficiency is often low, chemical and genetic modulation might help to promote the HDR pathway [92].

On the other hand, the edition of the endogenous BCR using the CRISPR/Cas9 system raises specific additional constrains, since the heavy chain needs to be specifically paired with a light chain to raise a fully active Ab. Indeed, if the light chain is not edited, chimeras of the modified heavy chain and the endogenous light chain will be obtained. Of concern, these chimeras might have a different specificity, recognize self-antigens, and lead to autoimmune reaction. In this respect, Lin et al. showed that the editing of only the heavy chain in ESCs impaired B cell differentiation, probably because of their elimination from the bone marrow owing to defective immunological checkpoints [89]. Thus, one obvious solution to reduce chimeras would be to target both the heavy and light chain loci. However, chimeras may still occur for two main reasons. First, due to the editing efficiency, all cells will not be edited at both loci. Second, only one allele coding immunoglobulin chain is normally expressed due a mechanism called allelic exclusion. Consequently, if the modification hits the productive allele, only the edited antibody would be expressed, whereas if the editing hits the nonproductive allele, both the endogenous and the modified chains would be expressed and chimeras might still be obtained. While the ideal solution would be to edit both alleles or to only edit the productive allele, another possible solution to avoid chimeric immunoglobulins and autoreactivity was developed by Moffett et al. upon physical linking of the light chain to the variable heavy chain on the donor DNA template, forcing the pairing of both the transgenic light and heavy chains [85]. Other strategies should be developed to force this pairing and thus, to avoid potential autoimmune responses. For instance, molecular engineering approaches have been created to promote T cell receptor ectopic chain assembling [93] and might be translated to the BCR. Alternatively, to avoid chimeras, a knockout of the endogenous BCR loci might be performed along with the insertion of the ectopic immunoglobulin sequence in another location. Nevertheless, with such a strategy, the regulation by the endogenous promoter can no longer be exploited and additional cuts are required, which increases the risk of off-target mutations.

The heavy chain locus contains several constant domains corresponding to different immunoglobulin isotypes. These subtypes exhibit various effector functions. Mature B cells initially express mostly IgM and IgD immunoglobulins, through an alternative splicing mechanism of the constant exons. During activation and differentiation, B cells undergo a process called isotypic class commutation. This process consists in genetic rearrangements of the heavy chain locus to remove proximal constant exons and kept distal constant exons (such as γ1, γ3, α1 corresponding to IgG1, IgG3 and IgA1 respectively). The activation-induced cytidine deaminase (AID) enzyme introduces cleavage in the heavy locus promoting genetic recombination. However, the susceptibility of a locus that is edited on its variable regions to undergo isotypic class commutation still has to be demonstrated. Interestingly, in 2018, Cheong et al. developed a tool to induce isotypic commutation by mimicking AID cleavages with the Crispr/Cas9 system [88]. They showed efficient commutation from IgM toward IgG. This study highlighted the feasibility of constant chain editing and paves the way for other modifications such as enhancing the immunoglobulin’s half-life or affinity to Fc receptors. Alternatively, Voss et al. showed that the ectopic variable antibody region can be targeted by AID for hypersomatic mutations, a process that enhances antibody affinity in the germinal centers, indicating that the construct must also be able to experience isotypic class commutation [87].

## 5. Conclusion and Perspectives

Over the past years, while the approaches to induce in vivo antibody secretion have greatly evolved, they currently only partially address the hurdles of passive antibody infusion. Passive antibody infusion presents several major drawbacks, such as the doses, the purity, the related adverse events and the costs of treatment. To circumvent these issues, gene delivery vectors have been developed to induce antibody secretion in vivo after in situ cell modification (Table 2). Initially, adenoviral and lentiviral vectors encoding antibodies were used to induce the expression of more physiological concentrations of antibodies, avoiding side effects associated to huge antibody doses. The expression of antibodies after vector infusion has proven to be effective in vivo in several cancer models. However, probably because of both the continuous expression of antibodies and their expression by unspecialized cell types, immune responses have been observed against the therapeutic antibodies. Furthermore, this permanent expression also promotes the appearance of escape mutants due to a high selective pressure. In this context, B cell therapy, particularly B cell adoptive transfer, has become an appealing solution. Indeed, B cells are specialized in antibody expression, both in a membrane-anchored and secreted form, and can now be easily engineered by vector transduction. Although B cells can be modified to constitutively secrete antibodies, several approaches aiming at regulated antibody secretion have emerged and could reduce both escape mutants and anti-idiotypic responses. One of the most promising concepts is the editing of the endogenous BCR with the CRISPR/Cas9 system in order to modify its specificity. With such an approach, the antibody secretion will be controlled by the endogenous regulation mechanisms avoiding or at least greatly reducing the above-side effects that are commonly experienced. Most cancers might be amenable for such approaches, including those forming solid tumors in which antibody infiltration is possible, but also hematopoietic cancers, except B cell lymphomas (since pathological B cells cannot be targeted without destroying the reprogrammed ones). In addition, similar strategies can also be applied to treat infectious diseases.

Overall, the capacity to genetically reprogram B cells could be extremely useful in order to elicit protective antibody responses against cancers. Ideally, this could be done by collecting patient B cells, modifying them before autologous re-infusion, though there remains major technical challenge to achieve this in an efficient manner. Alternatively, in situ genetic editing might become feasible in the coming years. Nevertheless, clinically relevant animal studies are still highly required to really address the efficiency and safety issues associated to the in vivo Ab secretion before translation to humans. For instance, a BCR transgenic mouse model can be used to study the impact of such reprogramming on the endogenous repertoire. In parallel, the physiological consequences of modified cell infusion as well as the long-term persistence of edited cells, their re-activation potential and their differentiation into memory B cells need to be addressed in both immunocompetent and humanized immune mouse models [94].

Although the field has greatly benefited from technological innovations such as viral vector engineering, several improvements are still required in order to achieve efficient B cell reprogramming. For instance, CRISPR/Cas9 cutting and HDR efficiencies need to be enhanced in order to modify both alleles without inducing mutations. Potential candidates toward this goal are p53 inhibitors. Indeed, the CRISPR/Cas9 system has been shown to promote p53 activation, leading to cell cycle arrest and hence, to a decrease of recombination efficiency [95,96].

In the future, cell or vector libraries might become available to trigger the secretion of multiple antibodies at the same time in vivo, which would allow concomitant targeting of different markers of cancer cells and enhance treatment efficacy by reducing epitope spreading [97,98]. In addition, depending on the TAAs expressed by the cancer cells of a given patient, the Ab clones selected for treatment might be personalized. Alternatively, the BCR construct might also be engineered to create chimeric B cell receptors with enhanced recognition and effector properties. Some promising results have already been obtained with synthetic receptors, surely leading the path for other advances in the coming years [99].

## Figures and Tables

**Table 1:** Characteristics of endogenous BCR editing systems.

Characteristics	Cheong [88]	Lin [89]	Voss [87]	Greiner [86]	Moffett [85]
Target cell	Human B cells	Mouse ESC	Human B cells	Human B cells	Human B cells
Cas9 delivery	Retroviral vector	RNP	Plasmid	RNP	RNP
Cutting heavy chain	Yes (1 cut in constant region)	Yes (2 cuts in D and J regions)	Yes (2 cuts in V and after J regions)	Yes (1 cut in V region)	Yes (1 cut after J and before constant region)
Cutting light chain	No	No	No	Yes (1 cut in κV region)	No
Cutting efficiency	N.D.	26–54.5%	N.D.	N.D.	72%
Sequences inserted	None	VH	VH	HC and LC	LC fused to VH
HDR efficiency	N.D.	0–50%	0.21%	8.5%	30%
Promoter	Endogenous	Exogenous	Endogenous	Endogenous	Exogenous
AID/class switching	?	?	Yes	?	?
Target	Endogenous	HIV	HIV	TNF-α	RSV

VH: variable heavy chain, LC: light chain, HC: heavy chain, RSV; respiratory syncytial virus, TNF-α: tumor necrosis factor alpha.

**Table 2:** Comparison of different approaches for ectopic antibody expression.

Approach	Infusion of Cells	Target Cell Type	Single Injection	Physiological Regulated	Immune Memory	Other Limitations	Refs
Passive immunotherapy (Ab injection)	None	None	No	No	No	Anti-idiotypic Abs Immune escape	[16,17]
In situ vectored gene transfer	None	Vector infected cells	Yes	No	No	Anti-idiotypic Abs Immune escape	[28,29,30,31,39,41,42,43,44,46,47,48,49,50,51,52,100,101,102]
Molecular rheostat approach	B cells	B cells	Yes	No	No	Random insertions Chimeras Effect on the endogenous response?	[75]
FAM2 technology	B cells	B cells	Yes	Yes	Yes	Random insertion Chimeras Effect on the endogenous response?	[72]
CRISPR-edited BCR	B cells	B cells	Yes	Yes	Yes	Off-targets HDR efficiency Chimeras Effect on the endogenous response?	[85,86,87,88,89]

## Appendix A. Antibodies to Restrict Vector Tropism to Tumor Cells in Gene Therapy

Although some virus-derived vectors, such as Semliki Forest virus, have a natural tropism toward tumors, those derived from measles virus, retroviruses and adenovirus can be engineered to specifically target tumors, which could reduce side effects due to systemic injection, especially by restricting transduced cell types. Several polypeptides have been used for vector surface-engineering, among which are antibodies and cytokines [100,101,103,104,105,106,107]. For example, antibodies targeting tissue-specific or tumor-specific markers (e.g., HER2) have been used to control vector tropism in vivo in breast tumors. Alternatively, vectors carrying nanobodies or antibodies direct against VEGFR2 were proven effective to respectively target tumor vasculature [102,104].

## Appendix B. New Tools for Genetic Modifications of B Cells

Lentiviral vectors have been widely used to introduce therapeutic transgenes into hematopoietic stem cells (HSCs), T or B lymphocytes in order to correct or treat hematopoietic disorders and cancers. Although after transgene integration into the host genome, long-term correction might be provided, until recently the transduction efficiency was relatively low for these cell types. Lately, the development of novel surface-engineered LV achieved through pseudotyping with RD114 cat endogenous virus, baboon endogenous virus or measles virus glycoproteins has strongly increased transduction efficiency as well as the range of target cells, including primary B cells that were notoriously difficult if not impossible to transduce [60,61,108].
